# Knee-Jerk in Early Tabes

**Published:** 1902-11-01

**Authors:** 


### KNEE-JERK IN EARLY TABES.

Notwithstanding the name "progressive locomotor ataxy" which has been applied to the disease generally known as tabes, it has long been recognised that ataxy is by no means always present, especially in its early stages. In this, the so-called pre-ataxic stage, the recognised diagnostic signs are loss of the knee-jerk, Argyll-Robertson pupils, lightning pains, gastric crises, ocular palsies, amaurosis, etc. Especial stress has been laid by English observers upon loss of knee-jerk, it being held that this is an almost necessary phenomenon of early tabes as well as of the more advanced stages of the disease, while increase of the deep reflexes, combined with tabetic symptoms, has been taken to point to general paralysis. Dr. Wilfred Harris,[1] however, has lately shown that this is not always the case. It is well known that in some cases of peripheral neuritis, such as are due to alcohol or diphtheria, the knee-jerks may be notably increased in the early stages, perhaps for three or four weeks, afterwards becoming lost, and he holds that there is no apparent reason why in some cases of tabes the initial stages may not also be similarly characterised by increase of the tendon reflexes, which increase may subsequently be followed by their disappearance in the progressive cases, while in others, in which the disease seems for many years at least to remain stationary, the increase of the tendon reflexes may also persist for lengthened periods. Out of 46 cases of which notes were taken in regard to this point, in 22, or nearly 48 per cent., both knee-jerks were either brisk or increased, while in only 41 per cent. were they absent. Weir Mitchell in America, and Erb in Germany, seem also to have had no doubt as to the possibility or even frequency, of increase of knee-jerk in early tabes. Many cases of early tabes are, says Dr. Wilfred Harris, undoubtedly wrongly diagnosed as incipient general paralysis on account of the existence of exaggeration of the deep reflexes. But increase of the knee-jerks ought not to decide in favour of general paralysis unless accompanied by more definite signs, as slurring articulation, tremor, fits, and mental impairment. Even the latter symptom, though usually remarkable by its absence in tabes, is certainly met with in certain cases of that disease; and it must be remembered that the two diseases are closely allied, that they may be concurrent in the same patient, and that both are frequently due to a common cause—syphillis—acting on the nervous system. The importance of not being misled by the retention of the knee-jerks is perhaps most markedly shown in regard to cases with paroxysmal visceral symptoms, such as gastric crises, which are often diagnosed as colic, gastritis, gall-stones, and the like. "Indeed, laparotomy has been frequently performed in such cases." When paroxysmal recurring attacks of indigestion are complained of, the possibility of tabes should always be thought of, and the state of the pupils should be examined, the knee-jerks and sensation, especially on the thorax and outer surface of the legs, should be tested, and inquiry should be made for shooting pains, bladder trouble, or diplopia. But the presence of knee-jerk must not lead one to negative tabes. Loss of light reaction in the pupil is undoubtedly the most constant and suggestive sign of tabes, but even it may certainly, though rarely, be met with apart from tabes or general paralysis. But this pupil sign, partial or complete, in conjunction with thoracic or ulnar analgesia, are probably the two earliest signs in the majority of cases of tabes, though it is no doubt true that shooting pains, gastric crises, etc., sometimes precede them and occur in the absence of any further sign of tabes. In testing the motility of the pupils, in addition to testing each separately, both pupils should be shaded and uncovered together so as to obtain the maximal stimulus of the direct and consensual light reflex together. If a pupil reacts consensually but not to direct light, it is not a case of reflex iridoplegia but one of blindness from retinal or optic nerve disease.

[1] Practitioner, October, 1902.

